# Transient Global Amnesia as the First Clinical Symptom for Malignant B-Cell Lymphoma with Central Nervous System Involvement

**DOI:** 10.1155/2015/191709

**Published:** 2015-07-07

**Authors:** Atif Zafar, Ghulam Ishaq Khan, Sahrish Abdin, Muhammad Taimoor Khan

**Affiliations:** ^1^Department of Neurology, University of Iowa Hospitals and Clinics, Iowa City, IA, USA; ^2^Columbia University, New York City, NY, USA; ^3^Aga Khan University Hospital, Karachi, Pakistan; ^4^West Virginia University, Morgantown, WV, USA

## Abstract

We present the case of an elderly male who was diagnosed with transient global amnesia (TGA), only to be diagnosed with B-cell lymphoma with central nervous system involvement a few weeks later. This is the first ever case reported in literature with lymphoma presenting as TGA. Literature review and pertinent points regarding high-yield imaging protocol for presumed TGA patients are discussed.

## 1. Case Report

We present an interesting case of a 70-year-old right-handed male with past medical history significant for hypertension and paroxysmal atrial fibrillation (PAF) who was brought to the Emergency Room (ER) after experiencing acute onset of confusion. He had been in his usual state of health when he went out to mow the lawn in the afternoon. An hour later, his wife found him staring at the lawn mower appearing confused. According to her, he repeatedly asked, “How did I come here?” and “What is going on?” He was able to identify his wife and family members and recognize his home. He did not report a fall, loss of consciousness, weakness, dysarthria, or facial droop. There was no evidence of incontinence or tongue bite at the time. His symptoms persisted for over two hours, prompting the ER visit.

When initially assessed via telerobot, there were no focal findings on neurological exam. Assessment was not consistent with a stroke; hence, Tissue Plasminogen Activator (TPA) was not offered. He was eventually transferred to the ER at University of Iowa Hospital and Clinics for further evaluation. On arrival, he was oriented to person, but not to time. He had difficulty recalling his date of birth. He continued asking questions such as “What happened?” or “Why am I here?” He appeared cheerful and in no acute distress. Word registration was intact; however, he did not recall any of the three objects after 3 minutes. Other language modalities like reading, naming, and repeating were intact. Comprehensive lab testing, including thyroid function test, blood count, comprehensive metabolic panel, and B12 levels, was within normal limits. At baseline, patient was retired, living at home with his wife. He had social alcohol intake of 2-3 beers/week and no illicit drug or tobacco use. Family history was positive for a sister with leukemia and heart disease in his parents. There was no family history of strokes, TIAs, amnesia, dementia, or other neurological conditions.

The patient's history and exam were most consistent with transient global amnesia (TGA). Imaging studies revealed normal CT head. Brain MRI with and without contrast was normal for any intracranial abnormalities. There was a small nodular heterogeneous nonspecific signal on T1 in the right occipital region possibly involving the cranium which was not considered anything serious at that time. He was admitted for observation with EEG done the next morning; while the patient was still demonstrating residual symptoms of amnesia, he was reported as normal for awake and sleep state. Clinically, the patient recovered completely 18 hours after the onset of symptoms. Comprehensive neurocognitive testing done prior to discharge showed some weakness in verbal anterograde memory, likely constituting a residual effect of TGA. Visual, memory, and other cognitive functions were within normal range.

**Figure 1:**
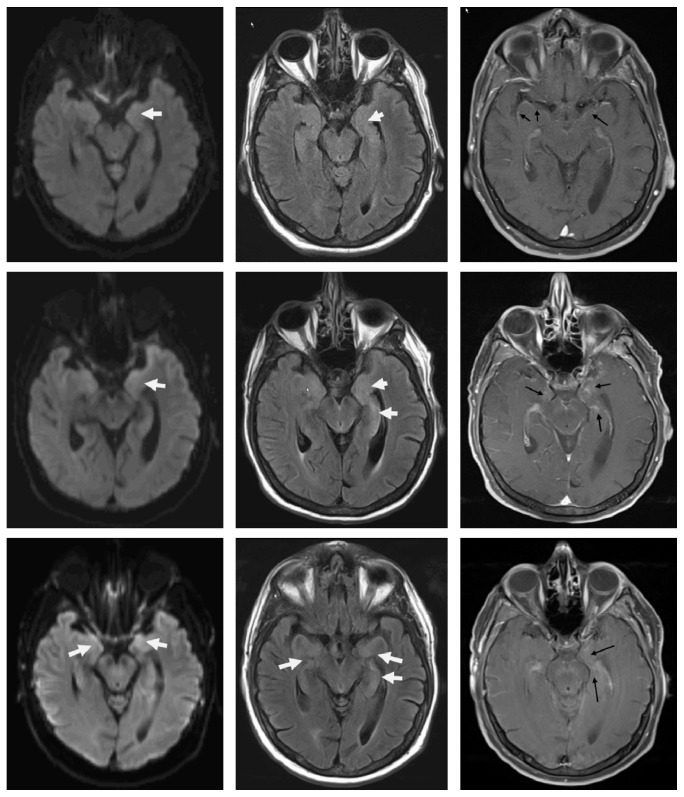
The first row shows brain MRI (from left to right, DWI, FLAIR, and T1+C) from first ER visit with presumed TGA: arrows show subtle signal changes that were too mild to be called. The middle row shows brain MRI (from left to right, DWI, FLAIR, and T1+C) six weeks from his first presentation: signal changes in DWI, FLAIR in hippocampus; moreover, there is enhancement in hippocampus and medial temporal lobe. The lowest row shows that brain MRI after treatment shows subtle improvement in hippocampus and temporal areas.

Two weeks after discharge, the patient's family reported that he has been sleeping half the day since he was discharged. Upon evaluation in the ER a few days later, there were no new findings on neurological exam. Six weeks after his first presentation, an urgent clinic appointment was set up with a brain MRI due to concerns of fatigue, progressive worsening of balance, cognition, and intermittent left sided weakness. Imaging showed nonnodular meningeal enhancement.  Figure 1 shows Magnetic Resonance Imaging (MRI) done at first encounter, comparing it to the sequences at six weeks and post-treatment. The patient was worked up with multiple CSF analysis, flow cytometry, CT scan, and bone marrow biopsy, leading to an eventual diagnosis of malignant B-cell lymphoma with spread to the CNS (subtype could not be classified). The patient underwent chemotherapy but expired 11 months after the diagnosis, 13 months from his first presentation with what was believed to be TGA.

Brain MRI performed on his first visit was discussed with neuroradiology, and it was concluded that he may have had subtle changes in the anterior temporal lobe and hippocampus on contrast and FLAIR scans, which became more pronounced with time. These changes were too subtle to be detected, and perhaps a 3 mm cut of the hippocampus would have been more high-yield.

## 2. Discussion

Transient global amnesia (TGA) entails acute onset of transient memory impairment (mostly anterograde but occasionally some associated retrograde amnesia) lasting less than 24 hours. It is mostly accompanied by repetitive questioning with an otherwise normal neurological exam [1, 2]. Theoretically, our patient fulfilled the diagnostic criteria for TGA (shown in Diagnostic Criteria for TGA (2)), despite the fact that the onset of symptoms was not witnessed. Posterior reversible encephalopathy syndrome was considered in the differential, since his systolic blood pressure was elevated in the 170s. However, the predominant deficits of anterograde (with some evidence of retrograde) amnesia and no signs of encephalopathy went against this diagnosis. Transient epileptic amnesia (TEA) (which involves brief, recurrent, witnessed episodes of transient amnesia with evidence of EEG abnormalities) was also in the differential, but there was no history of similar events in the past in the patient [3]. Moreover, spells in TEA are of shorter duration and occur multiple times instead of an isolated spell like in TGA. Additionally, EEG was reported as normal in this case.

Various phenomena to explain TGA have been described, with migraines and vascular, hypoxemic, and metabolic hypotheses being pointed out in various animal and imaging models, with general consensus on the amygdala and hippocampus as being the sites of involvement [1, 4, 5]. Our case, along with similar other publications, reemphasizes the role of the hippocampus and amygdala in amnestic presentations and the need to further investigate via imaging if there is a question about diagnosis of TGA in the ER [5, 6]. Less than two dozen cases have been reported where TGA was later identified as neoplastic or other similar pathological processes, mostly meningioma [5, 7]. Therefore, it may not be wrong to consider further imaging (i.e., MRI with thin DWI cuts) if the patient's presentation casts the slightest doubt on the diagnosis of TGA [8]. In this case, the spreading of B-cell lymphoma in the meninges especially in the hippocampus may have triggered a TGA-like event.

After retrospectively analyzing this reported case, we propose special protocolled thin hippocampal cuts for patients diagnosed with TGA [5, 9]. We understand that, for most patients in ER with a textbook presentation of TGA, further testing and imaging may not be feasible mainly due to the costs involved. However, for tertiary care centers that play a key role in advancing the field of neurosciences, we may be able to better understand amnestic syndromes like TGA if a brain MRI with and without contrast with 3 mm oblique axial hippocampal sections along the long axis of the temporal lobe in DWI and FLAIR films was to be obtained to improve the MRI yield. This, in turn, would benefit in the early diagnosis of possible secondary causes of amnesia (such as meningioma, metastatic tumors, and lymphomas) and prompt early treatment. According to our literature search, this is the first ever reported case of TGA-like presentation in a patient with malignant B-cell lymphoma.

Diagnostic criteria for TGA (2) are as follows:

 Attacks must resolve within 24 hours.
 Epileptic features must be absent.
 Attacks must be witnessed and information must be available from a capable observer who was present for most of the attack.
 There must be clear-cut anterograde amnesia during the attack.
 Clouding of consciousness and loss of personal identity must be absent and the cognitive impairment must be limited to amnesia.
 There should be no accompanying focal neurological symptoms during the attack and no significant neurological signs afterwards.
 Patients with recent head injury or active epilepsy (i.e., remaining on medication or with 1 seizure in the past 2 years) are excluded.

## References

[1] Bartsch T., Deuschl G. (2010). Transient global amnesia: functional anatomy and clinical implications. *The Lancet Neurology*.

[2] Hodges J. R., Warlow C. P. (1990). Syndromes of transient amnesia: towards a classification. A study of 153 cases. *Journal of Neurology, Neurosurgery and Psychiatry*.

[3] Butler C. R., Zeman A. (2011). The causes and consequences of transient epileptic amnesia. *Behavioural Neurology*.

[4] Choi B. S., Kim J. H., Jung C., Kim S. Y. (2012). High-resolution diffusion-weighted imaging increases lesion detectability in patients with transient global amnesia. *American Journal of Neuroradiology*.

[5] Milburn-McNulty P., Larner A. J. (2015). Transient global amnesia and brain tumour: chance concurrence or aetiological association? Case report and systematic literature review. *Case Reports in Neurology*.

[6] Alberici E., Pichiecchio A., Caverzasi E. (2008). Transient global amnesia: hippocampal magnetic resonance imaging abnormalities. *Functional Neurology*.

[7] Cianfoni A., Tartaglione T., Gaudino S. (2005). Hippocampal magnetic resonance imaging abnormalities in transient global amnesia. *Archives of Neurology*.

[8] Scheel M., Malkowsky C., Klingebiel R., Schreiber S. J., Bohner G. (2012). Magnetic resonance imaging in transient global amnesia: lessons learned from 198 cases. *Clinical Neuroradiology*.

[9] Ryoo I., Kim J. H., Kim S., Choi B. S., Jung C., Hwang S. I. (2012). Lesion detectability on diffusion-weighted imaging in transient global amnesia: the influence of imaging timing and magnetic field strength. *Neuroradiology*.

